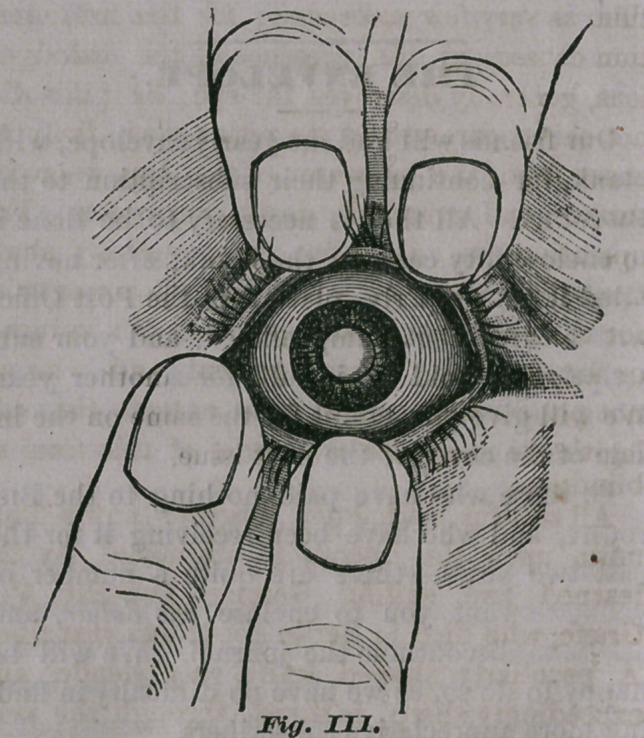# Cataract

**Published:** 1871-01

**Authors:** 


### CATARACT.

This is a term almost universally known, yet very few persons, even among the more intelligent classes of community, have any clear conception as to what cataract really is. Even the profession often exhibit most unpardonable ignorance in relation to this obstruction to vision, it being not an infrequent occurrence to have patients present themselves for operations for cataract, who had been recommended so to do by practicing physicians, when, upon examination of the eyes, nothing at all resembling such a condition of affairs, could justify their opinion.

Opacities of the cornea are frequently mistaken for cataract, and are generally so called by the people. It will be remembered that the transparent covering to the front of the eye, through which the pupil and iris are seen, is the *cornea*. This hard and clear coating of the eye, from its prominent and exposed position, is subject to wounds and inflammations, which is apt to be supervened by ulceration, in the healing of which, white or pearl-colored scars, or opaque spots are left upon the cornea, which interrupt the vision in proportion to their size and locality. Should a scar occur immediately over the pupil, it will prevent the passage of light, and produce partial or complete blindness, depending upon the size and density of the opacity. These scars are also called "films" or "pearls," and are sometimes caused by using patent eye waters, which always contain sugar of lead, and when applied to an ulcer upon the cornea, leaves a yellowish-white deposit of lead, which can never be removed. It is a generally accepted opinion that the ophthalmic surgeon can remove such spots surgically, but there exist very few, if any, opacities of the cornea that can be obliterated by any means.

In order to give a better understanding of what an opacity of the cornea is, we give below a very fair illustration of it:

*Fig. I.*

It will be seen that the scar is nearly round, and almost completely covers the pupil. A portion of the pupil may be seen below the obstruction, through which sufficient light would be admitted to enable the patient to see sufficiently to walk about. Now, in such an eye as this, nothing can be done toward *removing* the scar, but an operation can be performed for enlarging the pupil, (which we will describe in a future article), that would afford such a patient very good vision. But all this while we have been trying to explain what cataract is not, we hope to be more successful in describing what it really is.

Cataract is a term applied to an opacity of the crystalline lens, of its capsule, or of both. This lens is located immediately behind the iris and pupil, the light passing directly through the latter, next strikes the lens, whose function it is to refract the rays of light and bring them to a focus upon the retina, or nervous portion of the eye. This will be better understood by introducing another figure.

*Fig. II.*

We here have a section of the eye,—you are looking at it sidewise, and the eye is supposed to be cut half in two. The most prominent point to the right, is the cornea; the two dark lines, next behind, represent the iris, and the opening between them, the pupil; the oval body, marked A, is the lens. It will be observed that it is much larger than the opening in the iris—the pupil; therefore, if it should become white or opaque, no light would be able to reach the back part of the eye, for the opaque lens would completely shut it out, allowing it to progress no further than the pupil. Precisely this state of affairs exist in cataract. The lens becomes opaque, obstructing the passage of the light, and rendering the eye blind. A variety of causes exist for such an opacity. Wounds of the capsule or of the substance of the lens, from the prick of an awl, needle, knife or fork, as often occurs; blows from bats, balls or stones; falls from high places, which are apt to dislodge or displace the lens, are the principal causes for what the ophthalmic surgeon designates *traumatic* cataract,—to distinguish it from opacity produced by sickness, old age or natural causes. The disease known as diabetes often causes cataract. Inflammation of the internal structures of the eye, particularly of the iris, from rheumatism or syphilis, is apt to produce one form of the difficulty. But by far the most cases are the result of old age, designated *senile* cataract, where the opacity occurs from imperfect nutrition to the lens. It is by no means an uncommon occurrence to have children born with an opacity of the lens, then termed *congenital* cataract. The symptoms accompanying the approach of *senile* cataract, are at first a haziness of vision, as though looking through a fog. As the disease progresses, the flame of a lamp seems much enlarged, and luminous bodies are sometimes multiplied. The patient sees best at twilight, when the pupil becomes enlarged, allowing the ingress of more light, and permitting it to pass around the margin of the lens, where the opacity is not so great. Sometimes blindness becomes complete, or nearly so, (the patient being only able to see the light of a lamp, or tell where a window is), in a few month's time; in other cases several years elapse before the lens becomes hard and opaque. When the cataract is complete, the pupil appears grayish-white, or of a dull yellow color, caused by the diseased lens which is seen through it. It can be readily distinguished from a spot upon the cornea, by its being directly opposite the centre of the cornea, and *entirely* filling the pupil, as may be seen in Fig. III.

*Fig. III.*

By holding the hand over the eye for a few moments, in order to exclude the light, and then suddenly examining the eye again, the pupil will be found to be dilated, and the cataract will therefore appear proportionately enlarged. Traveling doctors take advantage of this fact, to swindle the patient by inducing him to believe that his cataract can be removed without the aid of surgery. They will drop a solution of atrophia into the eye, which in a few minutes will largely dilate the pupil, affording the patient considerable light, and perhaps some little vision, if the lens is not entirely opaque. This gives him some confidence, a handsome fee is paid the charlatan in advance, who soon decamps, leaving his victim as bad, or worse off, than before. No medicine, either locally or constitutionally, can be of any possible avail in removing an opacity of the lens. Many advertising charlatans profess to cure without the aid of a surgical operation, but their professions are false. There is but one means of relief, and that is with the knife, in the hands of a skillful operator. Years ago the "needle operation" was performed: A sharp and delicate instrument was thrust through the white of the eye, near the margin of the cornea, and passing behind the iris and pupil, either cut up the lens into small fragments, or depressed it, so that it would occupy a position behind the iris, and out of the way of the pupil, laying as represented at B, in Fig. II. The dotted outline shows the direction in which the lens is pushed. By this plan the lens, or its fragments, are left within the eye to become absorbed But this proved to be a very hazardous operation, as very few succeeded; for the inflammation caused by the presence of the dislodged lens, generally destroyed the eye; or if this did not occur, paralysis of the retina was likely to ensue from the pressure of the lens upon its surface, and eventually destroying vision. The operation has therefore, been entirely abandoned, except by a few surgeons who are anxious for the notoriety of an operation for cataract or for the money of the patient, and who do not possess sufficient skill to execute the only legitimate means for the relief of this form of blindness.

All skillful opthalmic surgeons now-a-days, unite upon the operation originated by the learned and skillful German Occulist, Von Græfe, who died in Berlin about one year since. A peculiarly shaped knife, very slender and exceedingly sharp, is thrust through the very margin of the cornea, very close to, and within the line where it is joined into the sclerotic, or white of the eye, and is carried across the front of the eye, behind the cornea and in front of the iris and pupil, and is made to come out in the same margin on the opposite side. A circular cut is then made, until nearly one-half of the cornea is cut loose from its margin to the sclerotic coat. An opening is thus made large enough to allow of the passage of the lens; the iris is next grasped by a delicate forceps, brought through the opening, and a small portion snipped out with the scissors; this enlarges the aperture in the pupil, and gives free passage to the opaque lens, which is next removed by rupturing the capsule that holds it in position, when, by slight pressure upon the globe, it floats forward to the opening in the cornea, where it is seized by a suitable instrument and removed from the eye. We now have a perfectly clear eye, and the patient is at once able to distinguish objects about the room. But the eye is not exposed longer than necessary to the light, as the retina, having been excluded from it so long, is sensitive, so that harm might be done by allowing the patient to use his eye at once. It is therefore carefully packed in dry lint and securely bandaged, so that no motion of the globe occurs. After two or three days, or after sufficient time has elapsed for the uniting of the wound made in the cornea, the bandage is removed and the light gradually admitted to the eye. In from four to ten days the wound is completely healed, and the eye able to bear the light, and be used for seeing purposes. It is now that art is brought to our aid, in supplying us with a new lens to take the place of the diseased one, removed from the eye. This is accomplished by means of the spectacle glass, of which two must be had, one for reading, sewing, or work where close scrutiny is required, and another pair for walking, or seeing at a distance. They are to be had at any first-class optical house, and are known as "cataract glasses," they being very powerful, double-convex-lenses, that cannot be used by others than those who have suffered the loss of the natural lens.

As surprising as it may seem to the general reader, yet is it a fact, that very little pain is produced by the operation just described. The incision is made through a tissue that is almost as devoid of nerves of sensation as the finger nail. It is very rarely the case that we administer an anæsthetic, and it is never necessary to hold the hands, or in any manner confine the patient, during such an operation, as very many people are in the habit of believing.

If we have succeeded in showing what an opacity of the lens really is; we hope our readers will cease calling every external blemish of the eye, or obstruction to vision, "cataract."

## Figures and Tables

**Fig. I. f1:**
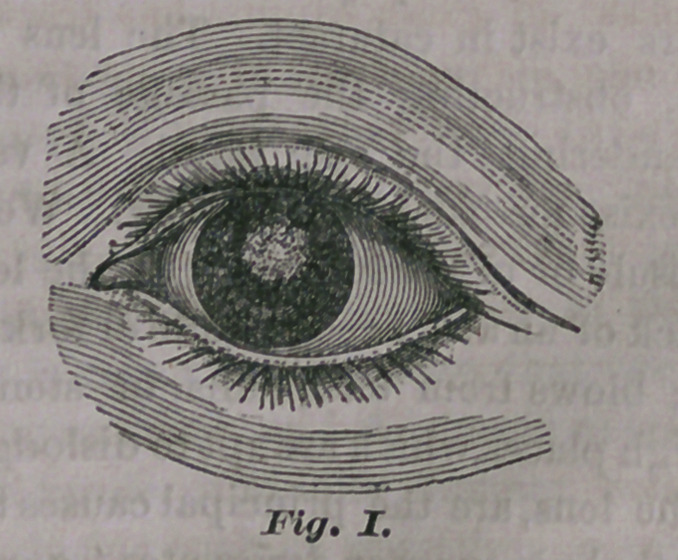


**Fig. II. f2:**
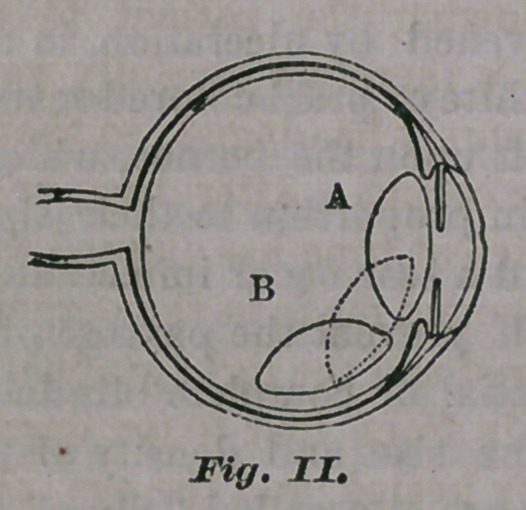


**Fig. III. f3:**